# Leisure time physical activity and exercise performance in active older people in rural areas–Comparison of the first and second COVID-19 related lockdown in Germany

**DOI:** 10.1371/journal.pone.0291560

**Published:** 2023-09-14

**Authors:** Maris Lohmöller, Tania Zieschang, Jessica Koschate

**Affiliations:** Geriatric Medicine, Department for Health Services Research, School of Medicine and Health Sciences, Carl von Ossietzky University Oldenburg, Oldenburg, Germany; Università degli Studi di Milano: Universita degli Studi di Milano, ITALY

## Abstract

The closure of all sports facilities during the two lockdowns in Germany favoured a reduction of leisure time physical activity during the COVID-19 pandemic. The aim of this study was to compare leisure time physical activity during the 1^st^ and 2^nd^ lockdown and to examine exercise performance before and after resumption of exercise. Leisure time physical activity was measured by the Longitudinal Urban Cohort Ageing Study (LUCAS) functional ability index and energy expenditure in the Minnesota Leisure Time Physical Activity Questionnaire. Participants’ exercise performance was extracted from a chip-controlled fitness circuit. Differences were tested for statistical significance using Friedman tests. 35 participants above 60 years were included from the Oldenburg area (20 women, 15 men, mean age and standard deviation 71±6 years). The decline in energy expenditure was higher during the 2^nd^ lockdown (1^st^ lockdown: Median -55.7 kcal^.^day^-1^, Q_0.25_−121.3 kcal^.^day^-1^, Q_0.75_ 132.9 kcal^.^day^-1^; 2^nd^ lockdown: Median -119.7 kcal^.^day^-1^, Q_0.25_−255.6 kcal^.^day^-1^, Q0.75−65.1 kcal^.^day^-1^; Friedman test: p<0.001, n = 35, *W* = 0.262). The time spent in the fitness circuit decreased from lockdown to lockdown as well as the number of participants exercising there. Intense activities were performed during the two lockdowns by only 7 and 3 participants, respectively, and were not resumed by two-thirds of the participants after the 2^nd^ lockdown. During the 1^st^ lockdown, exercise performance on resistance exercise devices increased in most of them, while it decreased by 1 to 7% during the 2^nd^ lockdown. The lockdowns limited leisure time physical activity in older adults. This was more pronounced during the lockdown in winter 2020/2021, when participants engaged less in outdoor activities. Therefore, measures should be taken to maintain physical activity and muscle strength, especially during winter months, with a home-based training, if visiting gyms is not possible.

## 1. Introduction

With onset of the COVID-19 pandemic in March 2020, many new legislative measures were introduced worldwide to contain the virus. These often included the closure of sports facilities and other public institutions [1]. Thus, the opportunity to engage in sports activities in leisure time was reduced to a minimum. Stockwell et al. observed in 64 of 66 studies included in their review a change of physical activity (PA) from November 2019 to June 2020 [2]. Within this period, a 1^st^ lockdown (LD) was installed in many countries. The majority of studies reported a decreased PA with a concomitant increase in sedentary behaviour [2, 3]. However, sporadically, an increase in overall PA or specific activities (e.g. walking, dancing) was observed [3–5].

The World Health Organization (WHO) recommends that adults engage in at least 150 to 300 minutes of moderate or in 75 to 150 minutes of vigorous aerobic PA per week [6]. During the 1^st^ LD, the proportion of those meeting the WHO-recommendations decreased from 38.1% to 30.4% in a German study [7]. Physical inactivity is associated with major long-term health damage that could be prevented [8]. Therefore, it is of interest to examine PA during the further course of the COVID-19 pandemic [9, 10], particularly during the 2^nd^ LD in the winter of 2020/2021.

Regarding the 2^nd^ LD period in Germany, only one online survey by Füzéki et al. (2021) is available, conducted in early summer 2021. There, transport-related PA decreased by 245.5 metabolic equivalent of task (MET) min^.^week^-1^ and leisure time PA by 52.7 min^.^week^-1^ [11]. Further studies on the 2^nd^ LD and beyond are lacking to date. Comparability between both LDs is complex for several reasons. During the 1^st^ LD, outdoor activities such as walking and bicycling were commonly used as alternative to sports activities [12]. In addition, overall PA appears to be higher in spring/summer than in fall/winter [13]. The 2^nd^ LD lasted longer than the 1^st^, hence the impact of closed sports facilities on leisure time physical activity (LTPA) may have been more dramatic during winter. We therefore assumed that people were less active in the winter LD than in the spring/summer LD. In addition, PA data during the pandemic LDs in Germany were almost exclusively assessed via (online) surveys and only few objective data are published to date [14, 15]. An analysis of objectively recorded muscle strength and endurance performance from the start of the 1^st^ LD until August 2020 showed a slight, but not significant improvement in both parameters in a small sample [14]. In contrast, Zieschang et al. found a decline in leg and rowing scores in the 1^st^ LD, affecting especially those who had trained at high intensity before the pandemic [15]. Both studies relied on exercise performances in a fitness circuit as objectively measured data, as was the case in this study. Altered LTPA during the LD, as observed in other studies, may have an impact on muscle strength and endurance. This might become visible in a changed exercise performance in the fitness circuit even if the exercise performance is sport specific. Muscle strengthening and/or endurance are required in many sports and are responsible for many negative consequences of non-exercising especially in older people, which leads to a reduction of muscle mass and muscle strength [16], increased risk of falling or frailty with consecutive higher mortality and morbidity rates [17]. In this context, the analysis of exercise performance seemed interesting, even though a possible change does not automatically have to be due to a change in overall PA.

In this study, we observed PA mostly in the leisure time, which according to the WHO includes both exercise training and recreational activities such as going for a walk or gardening [6]. According to Stockwell et al., many studies on this topic did not define the terms “exercise”, “PA” or “sports” to the participants [2], which leads to inaccuracies in the calculation of total PA because e.g. household-related activities or maintenance works at home probably would not be considered. We included household-related activities in the expression “leisure time PA” because it simplifies the recording of total physical activity, as e.g., gardening could also be seen as housework by the participants. Work related PA was not considered since we assumed that most participants were no longer employed.

The aim of this study was to analyse how LTPA of adults in the Oldenburg area has changed during the 1^st^ and 2^nd^ LD of the COVID-19 pandemic and to compare these data with objective training performance before the LDs and after training resumption.

## 2. Material and methods

### 2.1 Study design and procedure

The prospective cohort study was conducted from March 2020 until March 2022. Throughout this period, 8 to 9 telephone interviews were performed based on a questionnaire by 5 persons, all trained in advance. Due to the 2^nd^ LD, the plan to interview each participant after individual restart of exercise training and 6 months thereafter had to be adjusted accordingly. Table 1 shows the survey periods including brief explanations of governmental restrictions in Germany.

**Table 1:** Survey periods and corresponding government restrictions related to the COVID-19 pandemic in Germany.

Survey	Survey period	Description of current pandemic situation
1	04/23/2020–05/20/2020	pre-pandemic baseline (retrospective data collection for February 2020)
2		1^st^ LD in Germany: sports infrastructure closed since 03/16/2020
3	05/19/2020–06/30/2020	resumption of training after the 1^st^ LD
4	07/13/2020–07/14/2020	extra survey period for participants with later restart of training after the 1^st^ LD (n = 4), data replaced survey 3 of the corresponding 4 participants
5	11/30/2020–01/13/2021	follow up survey 5 months after the resumption of training (retrospective data collection for October 2020)
6		2^nd^ LD period in Germany: sports infrastructure largely closed since 11/02/2020
7	04/15/2021–05/03/2021	final phase of the 2^nd^ LD: training partly possible for a few weeks depending on regional incidence value
8	07/06/2021–07/28/2021	follow-up survey: end of all pandemic restrictions of the 2^nd^ LD
9	01/27/2022–03/08/2022	follow up survey: 6 months after restart of training

LD = lockdown; n = number of participants.

Ethical approval was obtained in advance by the ethics committee of the medical faculty at the Carl von Ossietzky University, Oldenburg (2020–061) and the study was registered at the German clinical trials registry (DRKS00021432). The study was performed in accordance with the 1964 Helsinki Declaration and institutional ethical standards of the University of Oldenburg.

### 2.2 Pandemic related restrictions in Germany

Restrictions due to the pandemic varied widely around the world. Therefore, the restrictions in Germany during the 1^st^ [18, 19] and the 2^nd^ LD [20, 21] are presented in Table 2. Restrictions were comparatively uniform across Germany during the 1^st^ LD, in contrast to the 2^nd^ LD. The regulations during the 2^nd^ LD refer to the federal state of Lower Saxony, as the participants were acquired there.

**Table 2:** Comparison of COVID-19 related restrictions of public life in Germany during both lockdowns.

	1^st^ lockdown	2^nd^ lockdown
period of restrictions	16^th^ March - 9^th^ May 2020	2^nd^ November 2020 - 8^th^ March 2021
shops	non-essential shops were closed, essential shops (pharmacies, banks, post offices etc.) remained open*	
body-related services	cosmetic and tattoo studios, hairdressers etc. were closed, medically necessary treatments remained possible*	November and December: body-related services were possible*, from January: only medically necessary treatments were possible*
recreational and cultural facilities	cinemas, theatres, pubs, clubs, leisure parks, zoos, gaming halls, restaurants etc. were closed	
	the use of playgrounds was prohibited	playgrounds were allowed to be used considering the permitted number of people and households
educational institutions	kindergartens, schools, universities, music schools etc. were completely closed	institutions were open first*, but closed from January on
sports facilities	all public and private sports were completely closed including swimming pools, gyms	using public sports facilities privately with other household members was partly possible, excluding gyms, swimming pools
	outdoor sports alone or with one other person was possible at any time	
religion	meetings in churches, mosques, synagogues etc. were prohibited, but e.g. funerals were possible with a restricted number of persons*	meetings in churches and similar institutions were allowed*
large events	were cancelled	
tourism	travelling in and to Germany was highly restricted, overnight stays were only possible for business travellers	
social contacts	going out in public was allowed alone, with members of one’s own household or with maximum one other person	going out in public was allowed alone, with members of one’s own household or with a varying number of other persons

*with strict regulations: These could include e.g. compulsory wearing of mouth-nose-protections in public, disinfection routines, keeping physical distance from others, limiting the number of persons per m^2^ in stores and institutions, tracing of personal contacts, visits to public institutions with appointments only, access only for vaccinated persons and/or persons with a negative COVID-19 test certificate.

The end of the 2^nd^ LD is indicated as 8^th^ March 2021, as this was when the first restrictions were eased [22]. However, many restrictions remained in place and could be strengthened again depending on regional incidence values. Violations of the regulations were punished with fines up to 25 000 euros.

### 2.3 Participants

Physiotherapeutic practices that offered training in a milon^®^ strength-endurance circuit 1 were identified online and contacted via telephone. The 3 practices in and around Oldenburg were offered a financial reward if they contacted their clients and gave them our contact information. If interested, potential participants contacted the Department of Geriatrics at the University of Oldenburg themselves.

Individuals above 60 years were included who were exercising in a milon^®^ strength-endurance circuit 1 and had completed at least one exercise session in February/March 2020. All participants took part voluntarily, provided informed consent prior to all interviews, and received a financial reward for the interviews. Due to the risk of infection during the pandemic, the interviews were conducted exclusively by telephone and the informed consent was thus obtained verbally only but documented by the interviewer in the participant file without witnesses. The consent form was made publicly available on the department’s homepage during the study course. If the participant preferred written consent forms, they were offered the option of sending it via mail or e-mail. Participants had the option of withdrawing their consent at any time. All data were obtained pseudonymized with a six-digit ID for each participant. The training data of all participants were exported pseudonymously, in accordance with the general data protection regulation of the European Union, from the chip-controlled fitness circuits (milon^®^ industries, Emersacker, Germany) by a data scientist at milon^®^ industries for each month. This data extraction was performed using a six-digit pseudonym different to the telephone interviews, so no personally identifying information was exchanged for the data transfer. Matching of the data reported in the telephone interviews and the fitness circuit data was only possible for members of the study team for the duration of the study. Afterwards, the coding list necessary for matching will be deleted. This procedure was approved by the data protection officer and the medical ethics committee of the University of Oldenburg (No. 2020–61) before the beginning of the study.

### 2.4 Variables and measurement

All included variables and questionnaires are listed in Table 3.

**Table 3:** Overview of included variables and questionnaires.

Topic	Included variables
Demographics	age, gender, height, weight, body mass index, marital status, children, living situation (building, area, social situation)
Professional background	highest level of education at school, training, current employment status
Health status	medical conditions with known diagnoses, use of medical aids, level of care, level of pain via Numeric Rating Scale, current health complaints, hospital stays, number (and type) of falls in the past 12 months or since their last interview
Health-related quality of life	German version of Short Form 12 Health Survey (SF-12) [23, 24]
Leisure time physical activity	Minnesota Leisure Time Physical Activity Questionnaire* [25, 26], LUCAS functional ability index [27]
Exercise training during the pandemic	resumption and frequency of training, reasons for training pause
Training performance data	heart rate, power, training duration on endurance devices (bicycle ergometer, crosstrainer), training weight, number of repetitions, sets on resistance exercise devices (leg extension, leg curl, chest press, abdominal crunch, back extension, seated rowing)
Pandemic-related issues	cancelled medical or therapy appointments, changed habits, fear of infection with COVID-19, fear of having relatives getting infected with COVID-19, current burden of the pandemic, most important reason for the current burden, current pandemic burden compared to the first wave, perception of easing of regulations, vaccination status

* Modifications: addition of new activities (housework, gymnastics, aqua aerobic) and splitting of activities (cycling and cycling with an electric bicycle, jogging and nordic walking) as of October 2020; addition of new activities (physiotherapy, videotherapy) as of March 2021.

During the first interview in April/May 2020, participants answered the questionnaire twice (once with respect to pre-pandemic baseline and once for the current LD period). The Minnesota Leisure Time Physical Activity Questionnaire (MLTPAQ) for October 2020 was completed retrospectively at the interview in November/December 2020. The MLTPAQ is a questionnaire to estimate the energy expenditure during leisure time, including household activities [25]. The energy expenditure of each activity is calculated as the product of its duration in minutes and its intensity code. By summing up energy expenditures of all listed activities, a total energy expenditure can be calculated which in this study served as a parameter indicating the participants’ LTPA. We used the shortened version (in German) of Fried et al. [26], which asked for the duration of these activities in the last two weeks unlike Taylor et al. who used the duration of exercise in the last year [25]. The following activities were queried (intensity codes in brackets, original activities of Fried et al. in italics): training in the chip-controlled fitness circuit (5.0), *going for a walk* (3.5), *mowing lawn* (6.0), *gardening* (4.5), *housework* (3.3), *hiking* (6.0), *cycling outside* (7.5), cycling with an electric bicycle (5.2), *bicycle ergometer* (4.0), *dancing* (5.5), *gymnastics* (3.8), *jogging* (6.0), Nordic Walking (4.8), *swimming* (6.0), aqua aerobic (5.5), sailing (3.0), *tennis* (8.0), badminton (7.0), *golf* (walking and carrying clubs) (5.0), active physiotherapy (3.8), videotherapy (3.8). In the course of the interviews, more activities with their respective intensity codes [28] were added, in order to assess all relevant LTPA (compare Table 3). Especially in observations with short recall periods, reliability for the original version is high (r = 0.79–0.88) [29]. Validity was found to be good in comparison with PA records and other questionnaires, but showed weaknesses when comparing to data retrieved from accelerometers [30, 31].

The Longitudinal Urban Cohort Ageing Study (LUCAS) functional ability index assesses 6 risks and 6 resources in activities of daily life [27]. Based on that, people can be classified as robust, post-robust, pre-frail or frail depending on their functional competence. The index is used to detect changes in functional status at an early stage of deterioration and correlates with the future need of nursing care and mortality.

The training performance data were analysed as follows: the last 3 training sessions before 03/17/2020 (1^st^ LD) and before 11/02/2020 (2^nd^ LD) were analysed as pre-LD data. Similarly, the first 3 training sessions after individual restart of training after both LDs were analysed for post-LD data. If 1 of the 4 time points (before/after 1^st^ LD, before/after 2^nd^ LD) was completely missing, the participant was excluded from the training data analysis for this exercise device (for detailed information on the number of excluded participants, see chapter 3.3). If only 2 or less training sessions per time point were performed instead of 3, the mean was calculated from the remaining data of each time point. A strength score was calculated for each resistance exercise device by multiplying the total (concentric plus eccentric) weight [kg] with the total number of repetitions. The endurance score was calculated by dividing the average heart rate [min^-1^] by the average power [W].

### 2.5 Statistical analysis

After the pseudonymously paper-based collection of the data, 2 persons transferred them independently into the REDCap electronic data capture tools and a third person corrected differences based on the original records [32, 33]. Data of the chip-controlled fitness circuit were prepared for analysis using Microsoft Access Version 2212 (Redmond, USA).

Initially, a sample size calculation showed that 100 participants should be included. Due to the complexity of recruitment described above, this could not be met. As a consequence of the small sample size, this study was no longer intended to generate statistically significant results, but rather to give a broad overview of LTPA of active seniors in northern Germany during the COVID-19 pandemic. Categorical variables are presented as frequencies, and metric variables as mean (M), standard deviation (SD) and 95% confidence intervals (95% CI) or in case of non-normal distribution as median (Med), 25^th^ (Q_0.25_) and 75^th^ quantile (Q_0.75_). Normal distribution was tested with the Shapiro-Wilk test. Differences in ordinally scaled or metric, non-normally distributed variables were tested for statistical significance using the Friedman test and, if significant, further examined using the Dunn’s post-hoc test with Bonferroni correction for multiple testing. Effect sizes were estimated using the Kendall coefficient of concordance (*W)* and were regarded as small (*W*<0.3), moderate (0.3≤*W*<0.5) or strong (*W*≥0.5). Normally distributed, metrically scaled parameters were analysed with analysis of variance with repeated measures, expanded by Bonferroni post hoc tests for pairwise comparison of statistically significant results. Partial eta square (η_p_^2^) is given for estimating effect sizes in this case. Significance was adjusted by Huynh-Feldt correction, if sphericity could not be assumed.

Participants with incomplete data were included as much as possible and excluded listwise for the Shapiro-Wilk test, the Friedman test and the analysis of variance with repeated measures. The number of participants analysed was therefore reported in each test result. Statistical analysis was performed using IBM SPSS software 28.0.1.0 (IBM Corporation, Armonk, NY, USA). Statistical level of significance was set to *p*≤0.05.

## 3. Results

### 3.1 Participants’ characteristics

35 persons participated in this study, of which 3 did not complete the last interview in February 2022 due to lack of interest. In hindsight, one participant was excluded from the training data analysis because of missing pre-pandemic exercise performance data. The majority was female, married or in a relationship and already retired. Detailed sample characteristics are shown in Table 4.

**Table 4:** Characteristics of the study population.

Variable		n
**sex**	male	15
	female	20
**age** [years]	mean (SD)	71.2 (6.3)
	95% confidence interval	69.0–73.3
** **	range	61–85
**body mass index** [kg^.^ m^-2^]	mean (SD)	26.5 (3.8)
	95% confidence interval	25.2–27.8
	range	18.8–36.0
**marital status**	unmarried	1
	married or in a relationship	28
	living separately	1
	divorced	1
	widowed	4^a^
**children**	yes	30
	no	5
**living situation**	house	30
	apartment	5
	rural area	24
	town	7
	alone	6
	alone with help (nursing service, domestic help)	1^b^
	with spouse	25^c^
	with spouse and help	3
	with other family members (between 16 and 30 years)	2
**highest level of education**	secondary general school^d^ (about 9 years)	9
	Secondary school^e^ (about 10 years)	11
	Academic secondary school^f^ (about 13 years)	14
	no information	1
**training**	apprenticeship^g^	22
	university	12
	none	1
**occupational situation**	dependent employed full-time/ part-time	2
	marginally employed, mini-job	1
	unemployed	1
	retired	30
	incapacitated for work	1
	(additionally) honorary office	3
**LUCAS index**	Robust	29
	postRobust	6

n = number of participants, SD = standard deviation.

^a^ one widowhood was newly reported in February 2022.

^b^ changed to living community with help in February 2022.

^c^ one participant newly reported living with other family members in February 2022.

^d^ lowest level of secondary education including Haupt-/Volksschule, 9. Klasse Polytechnische Oberschule: the graduation “Hauptschulabschluss” entitles to attend vocational schools.

^e^ secondary education at the Realschule: the graduation “Mittlere Reife” entitles to attend vocational schools, technical colleges or higher grades of academic secondary schools.

^f^ highest level of secondary education in Germany including the graduation forms “Abitur” and “Fachhochschulreife”: both entitle the student to attend university. ^g^ includes vocational school, Meisterschule.

The body mass index was stable during the course of the study (beginning of the 2^nd^ LD: M 26.5 kg^.^m^-2^, SD 3.8 kg^.^m^-2^, 95% CI 25.2–27.8 kg^.^m^-2^; end of the 2^nd^ LD: M 26.6 kg^.^m^-2^, SD 4.0 kg^.^m^-2^, 95% CI 25.5–28.0 kg^.^m^-2^, p = 0.567, η_p_^2^ = 0.02, n = 35).

Regarding functional ability, the majority remained Robust during the study period, as the minimum was 27 Robust participants in 2022. During the 1^st^ LD, 3 participants were classified as preFrail, compared to 6 participants at the beginning of the 2^nd^ LD. One participant was reported as Frail in the 2^nd^ LD and 2 as postRobust, respectively.

### 3.2 Health status

32 participants reported having health problems before the pandemic began. These are listed together with the changes in Table 5.

**Table 5:** The participants’ pre-pandemic health status and its changes during the COVID-19 pandemic.

Pre-pandemic health status				n
**Orthopaedic**	Osteoarthritis of the knee			9
	Osteoarthritis of fingers and wrists			7
	Osteoarthritis of the hip			6
	back problems (back pain, degenerative wear, spinal canal stenosis, spinal fusion)			11
	knee complaints (pain, previous surgery, meniscal damage)			5
	shoulder complaints (previous fracture or surgery)			3
	gait insecurity			2
**Cardio-vascular**	hypertension			9
	coronary artery disease/ previous coronary stenting			7
	atrial fibrillation, valvular heart disease, diabetes mellitus			3
	history of myocardial infarction, hypercholesterolemia			2
**Neuro-logical**	history of stroke, polyneuropathy			3
	multiple sclerosis			1
**Mental**	depressive symptoms			2
	post-traumatic stress disorder			1
**Other**	eye problems			4
	chronic bronchitis, thyroid disorder, vertigo			2
	sleep apnoea, interstitial cystitis, bladder weakness, reflux, gout, immune deficiency, autoimmune gastritis, soft tissue rheumatism, skin problems, loss of taste and smell after skull fracture, history of breast cancer, history of prostate cancer			1
	use of assistive devices (e.g. urinary catheter, toilet seat elevator)			4
	level of care			2
**Changes of the health status**		**1**^**st**^ **LD**	**2**^**nd**^ **LD**	**Follow-up**
**Orthopaedic**	worsening of symptoms/diseases	2	5	3
	increase in pain	8	8	3
	new complaints/diseases^a^	-	1	1
	limited mobility	1	1	1
	surgery	1 (planned)	1	1 (planned)
**Cardio-vascular**	improvement of symptoms/diseases^b^	1	-	2
	worsening of symptoms/diseases	1	-	-
	surgery (stent implantation)	-	2	1
	new complaints/diseases^c^	-	2	-
**Neuro-logical**	worsening of symptoms/diseases	-	1	-
	new complaints/diseases^d^	1	1	-
**Mental**	improved mental state	1	1	1
	deteriorated mental state	3	-	-
**Other**	new complaints/diseases^e^	4	3	8
	new level of care	-	1	-
	new degree of disability	1	-	-
	hospital stays	-	2	1
	more motivation for exercise	2	-	-

LD = lockdown, n = number of participants.

^a^ ligament tear, Carpal tunnel syndrome.

^b^ weight loss with improved hypertension during the 1^st^ LD, decreased blood pressure or HbA1c in summer 2021/winter 2022.

^c^ arrythmia, stress angina.

^d^ migraine, neuralgia.

^e^ dental/ urological treatment, chronic bronchitis/bronchial asthma, vaginal carcinoma, eye complaints (3x), bronchitis, gastric tumour, weakness, syncope of unknown cause.

In particular, orthopaedic diseases worsened during the pandemic according to the participants. For example, 5 individuals reported a worsening of pre-existing orthopaedic conditions, especially during the second LD, whereas only 2 individuals had perceived this during the first LD. Another major problem was an increase in pain which was constantly reported by 8 participants in both LDs. However, median pain levels of all participants were continuously between 2 and 3 points (pts) of Numeric Rating Scale throughout the pandemic. Overall, pain levels seemed to slightly decrease during the 1^st^ LD (during 1^st^ LD: Med 2.5 pts, Q_0.25_ 0 pts, Q_0.75_ 4.5 pts; after 1^st^ LD: Med 2.0 pts, Q_0.25_ 0 pts, Q_0.75_ 4.0 pts), whereas pain levels rather increased during the 2^nd^ LD (beginning of the 2^nd^ LD: Med 2.0 pts, Q_0.25_ 0 pts, Q_0.75_ 4.0 pts; end of the 2^nd^ LD: Med 3.0 pts, Q_0.25_ 1 pts, Q_0.75_ 4.0 pts, p = 0.668, n = 31, *W* = 0.022). The reported maximum of pain levels was also higher during the 2^nd^ LD (beginning: 10 pts, end: 8 pts) in comparison to the 1^st^ LD (7 pts).

### 3.3 Leisure time physical activity

**Fig 1:**
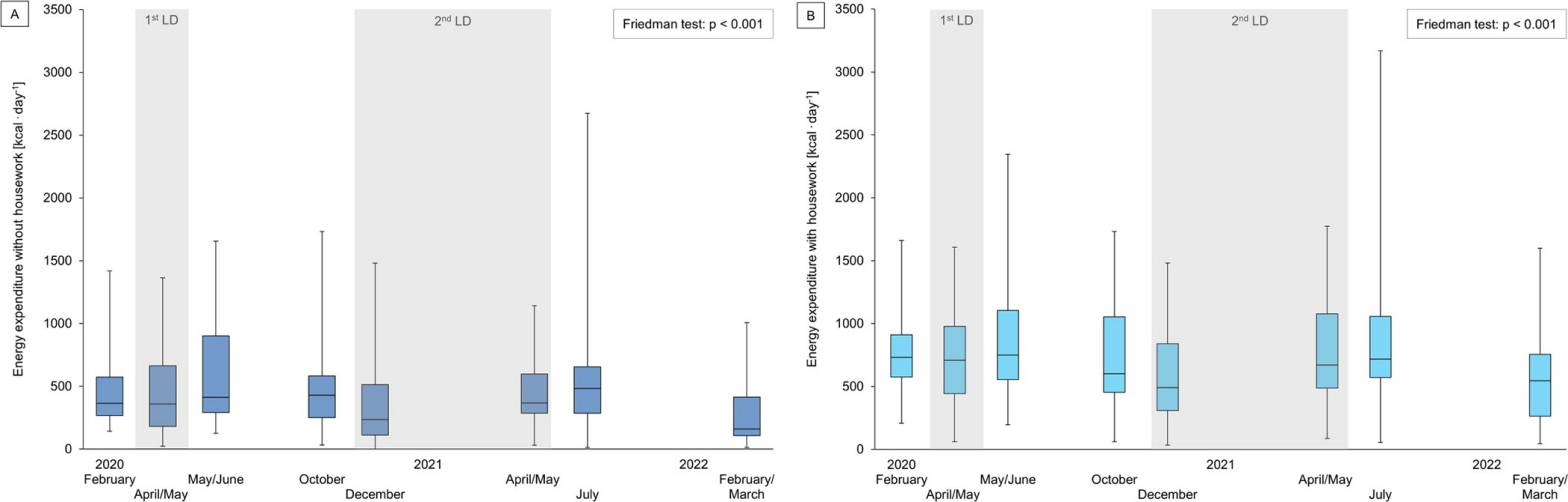
Total energy expenditure without (Fig 1A, dark blue) and with housework (Fig 1B, light blue). Housework as an activity was not recorded until October 2020. Therefore, the total energy expenditure (including housework) from February to June 2020 in Fig 1B was estimated based on the average kilocalories spent on housework thereafter. LD = lockdown, p = p-value.

Energy expenditure showed a similar trend during both LDs: The onset of public restrictions was followed by a decrease in energy expenditure, which was larger during the 2^nd^ than during the 1^st^ LD (1^st^ LD: Med -55.7 kcal^.^day^-1^, Q_0.25_−121.3 kcal^.^day^-1^, Q_0.75_ 132.9 kcal^.^day^-1^; 2^nd^ LD: Med -119.7 kcal^.^day^-1^, Q_0.25_−255.6 kcal^.^day^-1^, Q0.75−65.1 kcal^.^day^-1^, p<0.001, n = 35, *W* = 0.262). After easing of regulations, energy expenditure increased towards summer. The lowest energy expenditures were reported at the beginning of the 2^nd^ LD when including housework (Med 490.8 kcal^.^day^-1^, Q_0.25_ 308.1 kcal^.^day^-1^, Q_0.75_ 838.9 kcal^.^day^-1^, p<0.001, n = 32, *W* = 0.190), or without housework in winter 2022, even though there was no official LD (Med 160.3 kcal^.^day^-1^, Q_0.25_ 106.8 kcal^.^day^-1^, Q_0.75_ 414.0 kcal^.^day^-1^, p<0.001, n = 32, *W* = 0.255). Generally, energy expenditure was higher during the summer season than during winter. Since housework accounted for a considerable part of energy expenditure as of October 2020, energy expenditure was also calculated without housework (Fig 1A). Both calculations are shown in Fig 1 and follow an almost parallel course.

**Fig 2:**
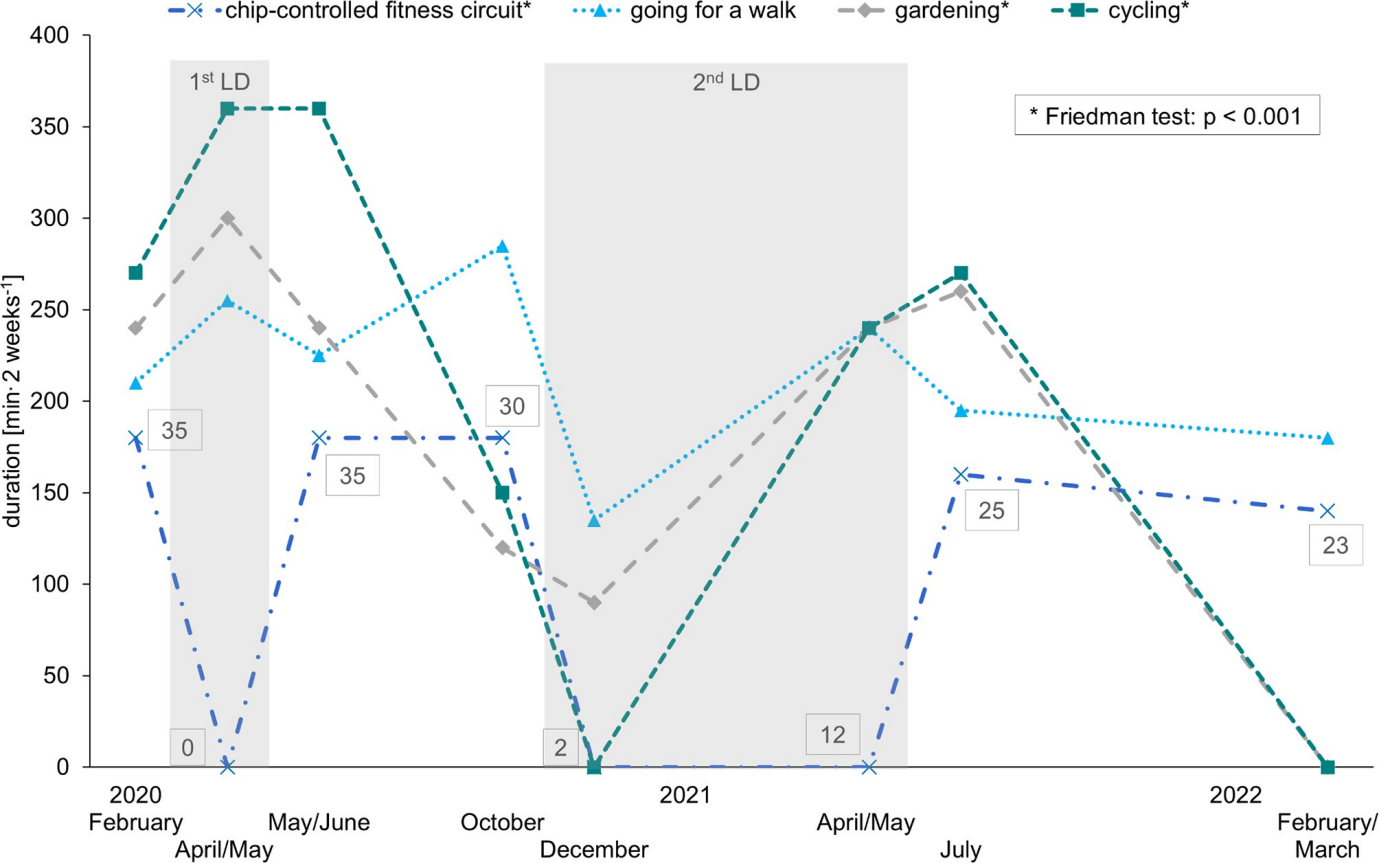
Median of the exercise duration of the 4 most popular activities during the pandemic. The numbers in boxes indicate the number of participants taking part regularly in the chip-controlled fitness circuit at each survey. Although 12 of the 32 participants have already returned to the fitness circuit in April/May 2021, the median of exercise duration is 0 minutes, as the remaining 20 participants have not yet started exercising again. Cycling includes summed times for outdoor bicycling and e-biking as of October 2020. LD = lockdown, p = p-value. For reasons of clarity, the 25% and 75% quantiles were not shown.

The most common activities at baseline were gardening, going for a walk, cycling outside and training in the chip-controlled fitness circuit. In the course, housework and cycling with the electric bike were also frequently mentioned. Badminton, sailing, tennis, and dancing were not performed by any of the study participants. The median time spent on the most popular activities is shown in Fig 2.

The pre-pandemic exercise duration in the chip-controlled fitness circuit was regained only after the 1^st^ LD. Beyond, the time spent exercising in the fitness circuit and the number of participants who exercised there regularly decreased. While all participants restarted training after the 1^st^ LD, only 5 out of 7 participants were still training after the 2^nd^ LD. In February 2022, reasons for non-participation in the fitness circuit were fear of infection (n = 2), health problems (n = 3) and too much effort due to the required COVID-19 test before a training session (n = 2). 3 persons terminated their fitness contracts, all in summer 2020.

Gardening and cycling developed in opposite directions during the two LDs: Time spent gardening or cycling increased clearly during the 1^st^ LD, but declined to pre-pandemic levels or below during the 2^nd^ LD. In comparison, gardening (1^st^ LD: Med 150 min^.^week^-1^, Q_0.25_ 7.5 min^.^week^-1^, Q_0.75_ 570 min^.^week^-1^; end of 2^nd^ LD: Med 120 min^.^week^-1^, Q_0.25_ 0 min^.^week^-1^, Q_0.75_ 300 min^.^week^-1^, p<0.001, n = 32, *W* = 0.305) and cycling (1^st^ LD: Med 180 min^.^week^-1^, Q_0.25_ 0 min^.^week^-1^, Q_0.75_ 337.5 min^.^week^-1^; end of 2^nd^ LD: Med 120 min^.^week^-1^, Q_0.25_ 0 min^.^week^-1^, Q_0.75_ 232.5 min^.^week^-1^, p<0.001, n = 32, *W* = 0.199) were performed longer during the 1^st^ LD than in the following year in April/May, when only a few restrictions were still in place.

**Fig 3:**
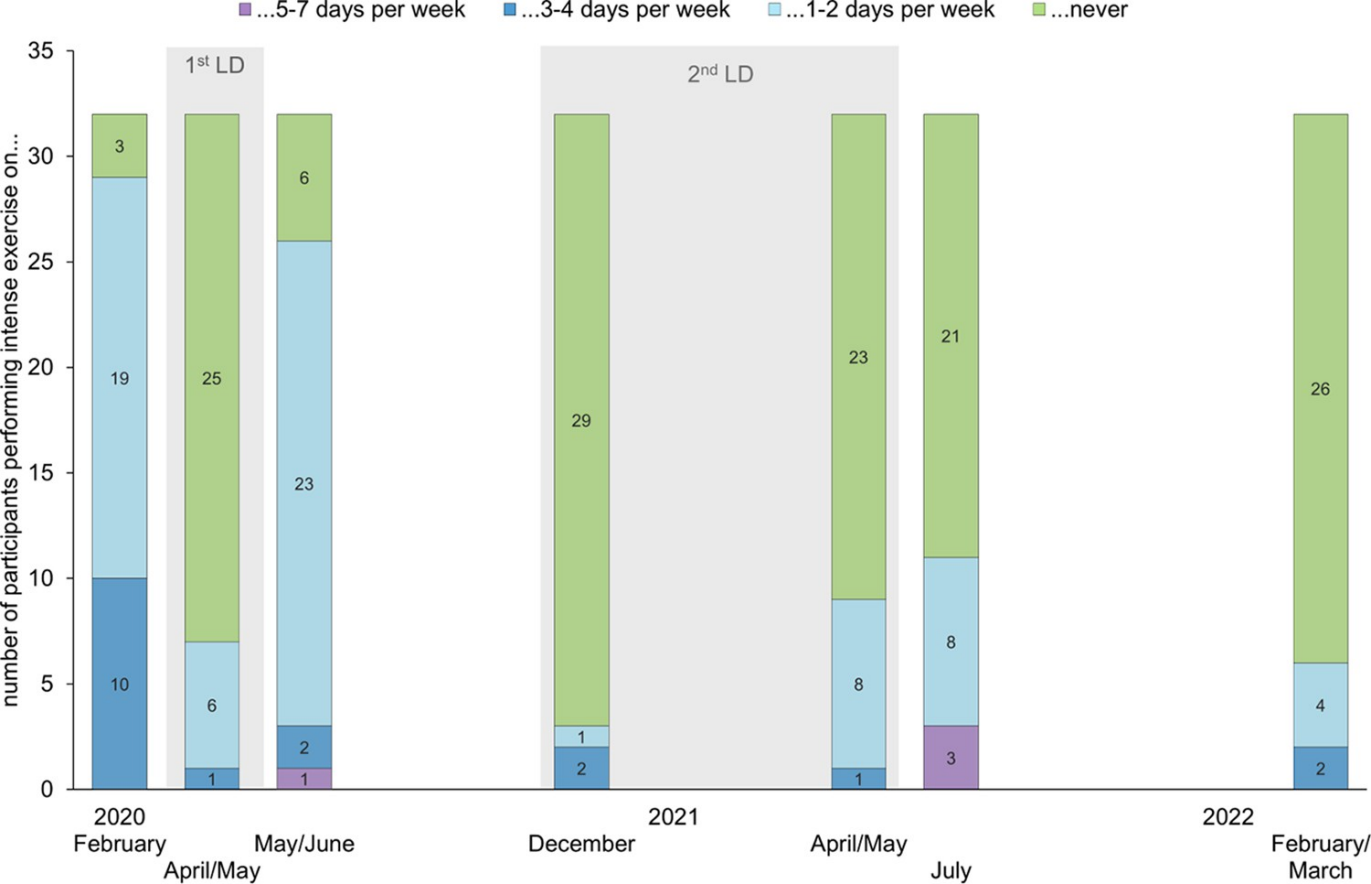
Frequency of performed intense exercise over the past 7 days during the pandemic. Intense exercise included e.g. jogging, sportive swimming or cycling. LD = lockdown.

The frequency of intense exercise, e.g. jogging, playing tennis or doing aerobics, has heavily changed during the LD periods, as can be seen in Fig 3.

Of 29 participants performing intense exercise on at least 1 day per week before the pandemic, only 7 participants were able to maintain this during the 1^st^ LD. From the beginning of the 2^nd^ LD, a large proportion of the participants no longer performed any intense exercise. Similarly, of all 35 participants doing moderately strenuous activities before the COVID-19 pandemic, only 28 participants and 22 to 25 participants, respectively, continued to do so during the 1^st^ and the 2^nd^ LD. In contrast, more participants reported walking outside on at least 3 of the past 7 days during the 2^nd^ LD (n = 26–29) than during the 1^st^ LD (n = 24).

To investigate the objectively measured physical fitness, the participants’ performance in the chip-controlled fitness circuit was analysed. For this analysis, 4 participants were excluded completely (one participant due to missing baseline data, the others ended their fitness circuit contracts in between the LDs). Depending on the exercise devices, incomplete data sets reduced the number of participants further. Results are presented in Table 6.

**Table 6:** Differences of strength and endurance scores within the COVID-19 pandemic.

		before 1^st^ LD	after 1^st^ LD			before 2^nd^ LD			after 2^nd^ LD					
device	n	median	median	*∆*	*Q*_*0*.*25*_*/ Q*_*0*.*75*_ *of ∆*	median	*∆*	*Q*_*0*.*25*_*/ Q*_*0*.*75*_ *of ∆*	median	*∆*	*Q*_*0*.*25*_*/ Q*_*0*.*75*_ *of ∆*	*#*	*Q*_*0*.*25*_*/ Q*_*0*.*75*_ *of #*	p *W*
		Q_0.25_/Q_0.75_	Q_0.25_/Q_0.75_	*∆%*		Q_0.25_/Q_0.75_	*∆%*		Q_0.25_/Q_0.75_	*∆%*		*#%*		
**chest press** [kg]	29	1150.0	1190.0	*23*.*3*	*-92*.*3/ 176*.*3*	1188.3	*86*.*7*	*-69*.*3/ 631*.*7*	1275.7	*149*.*3*	*-148*.*7/ 497*.*7*	*-73*.*3*	*-197*.*8/ 68*.*3*	0.140
		759.0/1463.5	834.2/1532.0	*2*.*0*		876.5/2287.0	*7*.*5*		930.0/1860.0	*13*.*0*		*-6*.*2*		0.063
**abdominal crunch** [kg]	28	1185.0	1187.7	*-14*.*8*	*-135*.*7/ 101*.*3*	1277.3	*15*.*2*	*-80*.*5/ 273*.*0*	1326.0	*47*.*8*	*-228*.*4/ 257*.*8*	*-92*.*2*	*-321*.*6/ 72*.*2*	0.279
		1031.5/1444.8	1037.9/1437.5	*-1*.*2*		1132.5/1627.8	*1*.*3*		1009.0/1453.0	*4*.*0*		*-7*.*2*		0.046
**leg curl** [kg]	29	1116.0	1190.0	*-53*.*3*	*-162*.*0/ 188*.*7*	1360.0	*80*.*7*	*-80*.*5/ 364*.*0*	1333.3	*52*.*7*	*-159*.*3/ 325*.*8*	*-16*.*7*	*-210*.*3/ 98*.*7*	0.146
		867.0/1676.0	956.0/1545.0	*-4*.*8*		990.5/1804.0	*7*.*2*		952.7/1809.5	*4*.*7*		*-1*.*2*		0.062
leg extension* [kg]	29	1222.7 (462)	1260.1 (441.6)	*37*.*5*	*-96*.*0/ 170*.*9*	1397.5 (495.4)	*174*.*8*	*-11*.*7/ 361*.*3*	1349.5 (465.2)	*126*.*8*	*-49*.*3/ 302*.*9*	*-48*.*0*	*-193*.*9/ 98*.*0*	0.078
		1046.9/1398.4	1092.2/1428.1	*3*.*1*		1209.0/1585.9	*14*.*3*		1172.5/1526.4	*10*.*4*		*-3*.*4*		0.078
back extension* [kg]	29	2186.4 (794.3)	2298.0 (806.3)**	*111*.*6*	*-108*.*9/ 332*.*1*	2600.8 (938.7)**	*414*.*4*	*109*.*5/ 719*.*3*	2457.4 (943.5)	*271*.*0*	*-34*.*9/ 576*.*8*	*-143*.*4*	*-364*.*2/ 77*.*3*	0.009**
		1884.3/2488.5	1991.3/2604.8	*5*.*1*		2243.7/2957.9	*19*.*0*		2098.4/2816.3	*12*.*4*		*-5*.*5*		0.140
**rowing** [kg]	29	1392.0**	1573.3	*24*.*0*	*-104*.*0/ 186*.*5*	1674.0**	*125*.*3*	*-81*.*3/ 280*.*5*	1629.3	*2*.*0*	*-106*.*8/ 427*.*7*	*-57*.*0*	*-216*.*2/ 27*.*0*	0.018**
		963.5/1698.7	984.5/1849.5	*1*.*7*		967.3/2412.3	*9*.*0*		1023.5/2074.8	*0*.*1*		*-3*.*4*		0.115
**crosstrainer** [min^-1.^W^-1^]	19	1.81	1.59	*-0*.*01*	*-0*.*10/ 0*.*35*	1.39	*0*.*01*	*0*.*44/ 0*.*24*	1.54	*0*.*0*	*-0*.*33/ 0*.*58*	*0*.*03*	*-0*.*08/ 0*.*20*	0.954
		1.16/2.86	1.31/3.28	*-0*.*6*		1.07/2.95	*0*.*6*		1.32/2.56	*0*.*0*		*2*.*2*		0.006
**bicycle ergometer** [min^-1.^W^-1^]	20	1.23	1.28	*0*.*08*	*-0*.*08/ 0*.*18*	1.23	*0*.*03*	*-0*.*07/ 0*.*29*	1.24	*0*.*08*	*-0*.*11/ 0*.*39*	*0*.*03*	*-0*.*06/ 0*.*22*	0.286
		0.99/1.42	0.97/1.48	*6*.*5*		0.96/1.54	*2*.*4*		1.02/1.55	*6*.*5*		*2*.*4*		0.063

LD = lockdown; n = number of participants with complete data for calculating the strength/endurance score on this device; ∆ = absolute difference to pre-pandemic median; ∆% = relative difference to pre-pandemic median in %; # = absolute difference to the median before the 2^nd^ LD; #% = relative difference to the median before the 2^nd^ LD; Q_0.25_/Q_0.75_ = 25^th^ quantile/75^th^ quantile; p = p-value; W = Kendall coefficient of concordance

* = normally distributed, mean (standard deviation) are presented instead of the median, 95% confidence interval instead of Q_0.25_/Q_0.75_ and η_p_^2^ instead of *W*

** = significant difference (between the marked time points). Interpretation: A negative sign indicates a deterioration in strength, but an improved performance in endurance devices. Calculation of strength scores [kg]: Strength score = (average concentric weight + average eccentric weight) * moves. Calculation of endurance scores [min^-1.^W^-1^]: Endurance score = average heart rate / average power.

For all resistance exercise devices apart from the abdominal crunch and the leg curl, the strength score increased until the beginning of the 2^nd^ LD. Afterwards, it decreased but remained up to 13% above the pre-pandemic level. This tendency was observed in all resistance exercise devices but was significantly different only when comparing strength scores after the 1^st^ and before the 2^nd^ LD for the back extension device (after 1^st^ LD: M 2298.0 kg, SD 806.3 kg, 95% CI 1991.3–2604.8 kg; before 2^nd^ LD: M 2600.8 kg, SD 938.7 kg, 95% CI 2243.7–2957.9 kg, p = 0.009, η_p_^2^ = 0.140, n = 29) and the rowing device at different time points (before 1^st^ LD: Med 1392 kg, Q_0.25_ 963.5 kg, Q_0.75_ 1698.7 kg; before 2^nd^ LD: Med 1674 kg, Q_0.25_ 967.3 kg, Q_0.75_ 2412.3 kg; p = 0.018, n = 29, *W* = 0.115). The biggest relative changes were observed for the back extension exercise device with 19% increase from before the 1^st^ to before the 2^nd^ LD. While the 1^st^ LD led to an increase in strength scores of 4 devices, these fell in all 6 devices by up to 7% within the 2^nd^ LD.

Endurance scores were calculated for only 19 to 20 participants because the belt for measuring heart rates was not always worn. An increase in endurance scores indicates a deterioration in physical fitness, as a higher heart rate was achieved for the same power. Endurance scores worsened on the bicycle ergometer after both LDs. In contrast, the 1^st^ LD led to a slight improvement on the crosstrainer, whereas a deterioration was observed in the 2^nd^ LD.

**Fig 4:**
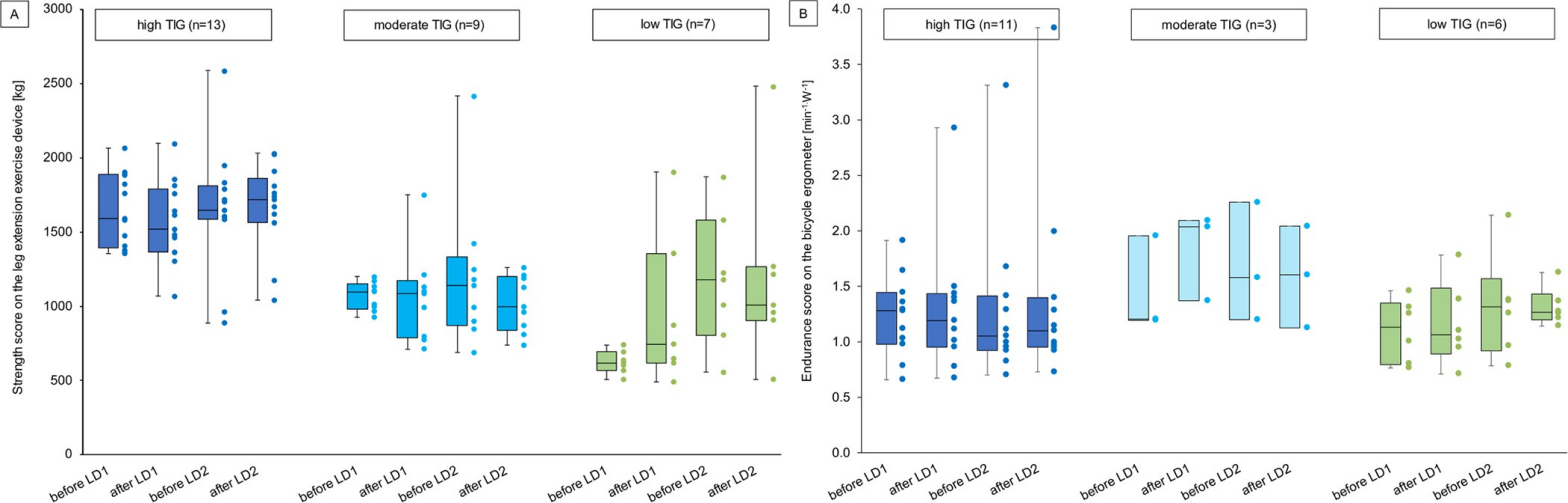
Exercise performance on the leg extension device (Fig 4A) and the bicycle ergometer (Fig 4B) by training intensity groups. An increase in endurance scores indicates a deterioration of physical fitness. TIG = training intensity group; LD1 = 1^st^ lockdown, LD2 = 2^nd^ lockdown, n = number of participants. Cut off values for TIGs: high TIG: > 1324.33 kg, moderate TIG: 816–1324.33 kg, low TIG: <816 kg. Due to the small group sizes, individual values are shown as coloured dots next to the boxplot. In Fig 4B, only 3 participants of the moderate TIG had a complete data set (light blue boxplots). Thus, the 25^th^ quantile and the 75^th^ quantile cannot be calculated. Instead, the minimum and the maximum are presented.

According to the cut off values by Zieschang et al. [15], participants were divided into different training intensity groups (TIG) based on their preCOVID-19_leg score_. In this sample, 13 participants belong to the high TIG, 10 participants to the moderate TIG and 8 participants to the low TIG. Due to the small group sizes, the analysis here was limited to a purely descriptive approach. Their observation that especially the high TIG was affected by the interruption during the 1^st^ LD [15], was observed in the leg extension device here too. In contrast, the low TIG improved their strength score during the 1^st^ LD and beyond, but deteriorated during the 2^nd^ LD. Regarding endurance values, the 1^st^ LD led to a decline of endurance scores in the high and the low TIG, hence both groups improved their endurance capability. In the 2^nd^ LD, however, only the low TIG was able to improve their endurance capability. Fig 4 presents the results of this analysis.

### 3.4 Mobility in daily life

At the end of the 2^nd^ LD, 14 participants reported being slower than before or recently using assistive devices when walking, climbing stairs or getting on/off transport. In contrast, this was reported by only 3 participants during the 1^st^ LD. Due to fear of falling, 3 and 7 participants, respectively, limited certain activities during the 1^st^ and the beginning of the 2^nd^ LD. The average number of falls per month evolved from 1 fall per 2 months (1^st^ LD: Med 0, Q_0.25_ 0, Q_0.75_ 0, total number of falls: 1 in March/April 2020) to 1 fall per month (end of the 2^nd^ LD: Med 0, Q_0.25_ 0, Q_0.75_ 0, total number of falls: 4 from January to April 2021, p = 0.016, n = 32, *W* = 0.081). Common causes for the 22 to 23 falls during the course, were falls from bicycles (n = 5), at home (n = 3) and overlooked steps/curbs or staircase falls (n = 4). Affected participants each had a maximum of 2 falls between each survey, with the exception from the 2022 survey where one participant had 4 to 5 falls.

### 3.5 COVID-19 pandemic and health-related quality of life

The participants’ daily life was greatly affected by the restrictions imposed to prevent the spread of SARS-CoV-2. Some aspects are presented in Table 7.

**Table 7:** Impact of the pandemic on emotional state, behaviour and health-related quality of life.

			1^st^ LD		2^nd^ LD		p W
			Apr/ May ’20	May/ June	Dec ’20	Apr/ May ’21	
I am worried about **getting sick** with COVID-19.* [median, Q_0.25_/Q_0.75_]			3	2	2	2	0.002
			1.25/3	1/3	1/3	1/3	0.110
I am worried that people close to me will get sick.* [median, Q_0.25_/Q_0.75_]			3	3	3	3	0.012
			2/4	2/3	3/4	2/3	0.087
I cancelled a **medical or therapy appointment**.			13	1	1	1	
Medical or therapy appointments were cancelled.			14	5	6	1	
The restriction of social contacts burdens me the most.			25	20	21	19	
I have **changed my habits**:			-	-	19	13	
	being at home more often/ being less in public				4	5	
	having fewer social contacts than usual				9	7	
	going shopping less often				5	1	
	being outside and being more active				1	4	
Some aspects were **more burdensome** in the 2^nd^ LD					20	24	
	lack of perspective				2	6	
	high incidence of COVID-19 and related deaths				4	4	
	annoyance with unreasonable people				4	3	
	less outside activities due to worse weather				4	-	
	continued duration				1	7	
	restriction of social and public life				7	5	
	unclear government concept				2	4	
	occurrence of mutations of the virus				-	3	
	problems with organisation of vaccinations				-	2	
Some aspects were **less burdensome** in the 2^nd^ LD					11	14	
	habituation effect: wear face masks, keep distance				3	7	
	less fear and worries				2	5	
	more hope through existing vaccination				-	3	
	better reporting/ information transfer				4	-	
	no food shortage				2	-	
	LD was less strict (more personal freedom)				2	2	
**Health related quality of life**							
	Physical component summary score [median, Q_0.25_/Q_0.75_]		48	47	49	50	0.755
			41/54	40/54	34/55	44/54	0.018
	Mental component summary score [median, Q_0.25_/Q_0.75_]		56	58	56	58	0.085
			52/58	55/61	52/60	53/60	0.058
	I felt sad during the past 7 days.** [median, Q_0.25_/Q_0.75_]		5	5.5	5	6	0.006
			4/5	5/6	4/6	5/6	0.095
	Health problems affected my contact with others.*** [median, Q_0.25_/Q_0.75_]		5	5	5	5	0.041
			4/5	5/5	4.25/5	3.25/5	0.069

LD = lockdown; Q_0.25_/Q_0.75_ = 25^th^ quantile/75^th^ quantile; p = p-value; W = Kendall coefficient of concordance; Dec = December; Apr = April; ’20 = 2020; ’21 = 2021. Unless otherwise stated, the number of participants is indicated. *Answer categories: 1 = not at all, 2 = hardly, 3 = a little, 4 = very much.

** Answer categories: 1 = always, 2 = usually, 3 = quite often, 4 = sometimes, 5 = rarely, 6 = never.

*** Answer categories: 1 = always, 2 = usually, 3 = sometimes, 4 = rarely, 5 = never.

While the fear of getting sick oneself decreased over time, the participants were more worried about people close to them getting sick. Only one person was infected with COVID-19 (in summer 2021). By August 2021, 34 participants had been vaccinated (32 participants already twice). The end of restrictions in summer 2021 was perceived rather positively by 29 participants and rather negatively by 6 participants. Regarding health-related quality of life, mental and physical component summary scores did not show significant differences during the study period. However, it was striking that in both LDs, 10 participants felt sad which is 2.5 times as many as after the LDs/ at their end (4 participants).

## 4. Discussion

In this cohort study with 35 participants from the Oldenburg area, we analysed their LTPA and training performance in a chip-controlled fitness circuit during the 1^st^ and 2^nd^ LD of the COVID-19 pandemic in Germany and beyond.

The energy expenditure decreased during both LDs, with a greater extent during the 2^nd^ LD, and increased after easing of restrictions both times. This supports our hypothesis that the winter LD had a stronger impact on LTPA than the 1^st^ LD in spring, even though effect sizes were small in our study. The finding of a decline in LTPA in Germany due to a LD is in line with the current literature [7, 11] and was also observed internationally [2, 34]. Specifically for seniors above the age of 60 years, Oliveira et al. reported a decrease in PA levels as evidenced by increased time for sitting, a reduction in number of steps and of MET values [34]. In contrast to Oliveira et al., our study participants reported spending more time walking during the 1^st^ LD, but a direct comparison is difficult because we did not measure the number of steps with an accelerometer. Presumably, the real duration of walking during the pandemic is even higher than indicated in our study, since light and moderate-intensity activities in particular tend to be underestimated in the MLTPAQ in contrast to high-intensity activities due to recall errors, as validity studies have shown [30]. However, the contrast between the results may be explained by the different restrictions applying in distinct countries because the respective studies were carried out in Brazil, China and Japan [35–37]. In all these countries, activities outside one’s home were strictly regulated while there were no restrictions for outside activities as walking and cycling in Germany. Regarding the further course of the pandemic, our results are in accordance with those of Oliveira et al. [34].

Since the energy expenditure was even lower without a LD in the winter of 2022, the decrease in December 2020 may partly be explained by seasonal influence. At the same time, participants may have voluntarily maintained their pandemic-adapted behaviour over the winter of 2022, e.g. due to the high number of COVID-19 cases in Germany [38]. Gardening and cycling showed clear seasonal differences with higher popularity in spring and summer. Therefore, both served as alternatives to sports activities mainly during the 1^st^ LD, which has been observed in other studies as well [12, 39]. Gardening for example showed a significant, moderate effect during the study course: it was done 30 minutes more per week in April/May 2020 than in the following year, when the 2^nd^ LD was ending and first restrictions were eased. Most participants did not cycle during the winter LD, which did not differ from winter 2022 without LD. Training in the chip-controlled fitness circuit was performed most before outbreak of the COVID-19 pandemic and lost importance from LD to LD. Whereas all study participants restarted exercising after the 1^st^ LD, only 5 out of 7 remained training after the 2^nd^ LD. This effect was also seen in the frequency of performing intense exercise, as Fig 3 presents. Hence, the drop in LTPA, caused by a LD, seemed to last longer after the 2^nd^ LD than after the 1^st^ LD. However, the statement on intensive activities must be worded cautiously, because participants apparently did not perceive the fitness circuit as strenuous activity. According to Figs 2 and 3, 23 persons were exercising on the fitness circuit in February 2022, but only 6 persons reported doing strenuous activities at least once a week at that time. Maybe, the participants perceived certain activities differently than stated in the literature which leads to an underestimation of LTPA in this case. Probably, the different understanding of “physical activity”, “exercise” and “sports”, as described by Stockwell et al. [2], further supports this inconsistency.

Regarding objective training performance, strength scores increased slightly over time in comparison to the pre-pandemic scores. However, a decrease of up to 7% was observed in all strength scores after the 2^nd^ LD while they increased mostly by maximum 5% after the 1^st^ LD. This finding further supports our hypothesis that PA was lower during the winter LD, even though only the rowing and the back extension exercise device showed statistical significance with a small effect. Endurance scores also increased comparing after to before both LDs, meaning the participants’ physical condition has deteriorated, with exception of the 1^st^ LD on the crosstrainer.

The different development of strength scores during the 1^st^ and the 2^nd^ LD could be explained by the prolonged duration of the 2^nd^ LD. Whereas endurance can be maintained easily in everyday life, by for example longer walks, muscle strengthening training is more difficult to replace. Therefore, a 6-month LD period probably resulted in a larger muscle loss than the 2-month LD period did. A decline in muscle-strengthening activities was also observed by Füzéki et al. during both LDs [7, 11]. Therefore, exercises with one’s own body weight or alternatives such as elastic bands should be promoted more strongly.

It is important to consider the participants’ age when using the different length of the LDs as an argument. On the one hand, the functional status can deteriorate rapidly within days by acute events, such as surgery. On the other hand, functional decline can progress slowly due to chronic diseases or as part of the normal ageing process, making detection much more difficult. Considering the participant’s age ranging from 61 to 85 years, a decline in functional status and exercise performance due to natural ageing are likely during the study period. It could be argued that this effect played a greater role during the longer observation period (2^nd^ LD) and therefore was at least partly responsible for the deterioration of the scores in comparison before and after the 2^nd^ LD. As absolute strength scores increased throughout the comparison from before the 1^st^ LD to after the 2^nd^ LD, this effect seems negligible.

Another explanation why strength scores rather increased during the pandemic even though energy expenditures declined in the LDs could be found in the reopening of do-it-yourself stores. Those were initially closed during the 1^st^ LD but reopened in the beginning of April 2020 which was much earlier compared to other stores (May/June). During the 2^nd^ LD, they were closed between December 2020 and March 2021, but online shopping was still possible in between. Thus, many people used the time for renovation of their homes and similar projects which might not have been reported as activity in our study. Home repair as activity also showed low validity in the MLTPAQ when comparing with PA records [30] and was not explicitly asked as an activity in our study. The role of do-it-yourself stores as described above was also seen as explanation of increased moderate PA during the lockdown in Poland by Czyz et al. [40]. For better estimation of this effect, precise data about home maintenance works would have been desirable.

Interestingly, the low TIG improved the leg extension strength score during the 1^st^ LD whereas the other TIGs worsened slightly (Fig 4), and also total energy expenditure of all participants decreased in both LDs. It seems unlikely that the low TIG started exercising intensely at home during the 1^st^ LD, when possibilities to exercise were heavily reduced. It therefore can be assumed that this incline of the low TIG in the 1^st^ LD is due to more activities in the light or moderate intensity domain than before the pandemic, e.g. doing home repair work, which was insufficiently reported as activity in the MLTPAQ. This argumentation is supported by several studies that observed an incline in moderate PA and walking in leisure time during the pandemic [12, 39–41]. This might have positively influenced muscle strength of the low TIG. However, these activities were apparently not enough to maintain the muscle strength of the high TIG, since their strength score deteriorated in the 1^st^ LD.

The subjectively perceived bigger burden of the 2^nd^ LD could indicate why only a few participants restarted exercising during/after the 2^nd^ LD. Due to the LD situation lasting for more than 6 months, participants complained about a lack of perspective. This may have led to a lack of motivation to restart engaging in sports activities. The effort to get tested for COVID-19 before going to the gym and the fear of infection during exercise may have deterred others, as the compulsory wearing of a face mask waived during exercising on the devices, although it was mandatory almost everywhere in public [20]. However, 29 participants perceived the loss of restrictions in summer 2021 rather positively but were afraid at the same time of easing the restrictions too fast. Apparently, concerns about a new LD were present. In contrast, wearing face masks, keeping distance, and having fewer social contacts had already become a routine for many study participants. Probably, not doing sports also became a routine after 6 months of constantly changing exercise options. This habituation to non-sporting is more likely after the long 2^nd^ LD than after the comparatively short 1^st^ LD in Germany.

One major strength of this study is the cohort study design which allowed prospective observation of the same participants and their PA throughout the pandemic, unlike other cross-sectional studies. It also had the advantage of short recall periods, increasing the validity of the MLTPAQ. Besides, PA measured by combining subjectively answered surveys and objective exercise data has rarely been examined even though the combination of the two can probably capture LTPA better. Data from a chip-controlled fitness circuit may better reflect overall physical condition than studies based on step count analysis, as they cannot consider muscle strength as a part of physical fitness. The inclusion of older people is another strength because they have often been underrepresented in (online) surveys.

An important limitation of this study is the small number of participants which results in low statistical power. The study population is not representative because regular participation in the fitness circle was required according to the inclusion criteria which does not account in general for the population above the age of 60 years. It can be expected that the participants of our study are more aware of the benefits of physical activity than most of the other older people because they are used to exercising regularly, maybe in order to combat physical complaints. This may have led them to pay more attention to being physically active during the pandemic than others, which may have positively influenced our results, even though only small to moderate effects were found in this study. Another source of inaccuracy could be the retrospective collection of baseline data and the addition of new activities to the MLTPAQ in the course. In case of housework, leading to increased levels of energy expenditure, it was corrected mathematically, hence the influence is negligible. However, it is known from validity studies that the longer the period of recall, the more difficult it is to remember irregular activities such as walking or household activities [30]. Regular appointments such as sports are easier to recall, which could have biased the comparison of the individual activities [30]. It is not apparent from the training data, how much the participants strained while performing the exercises. Therefore, we assumed that this was roughly similar for each training session. Questionnaires in general may have the limitation of overestimated results and low validity, but the MLTPAQ used for our study rather has a problem of underestimating certain activities [30]. This seems more plausible when considering the objectively measured data of the fitness circuit showing not as much decrease as we were expecting based on other study results [2, 7, 11, 15, 34]. They rather indicate that some other activities have contributed to the increase of strength scores which were not reported in the MLTPAQ. In addition, the combination of questionnaires with objectively measured data was chosen to avoid further limitations that can occur when using questionnaires.

## 5. Conclusion

In conclusion, LTPA, measured by energy expenditure and frequency of intense exercise per week, decreased clearly with the beginning of both LD periods in Germany. This effect is known from other national and international studies. Restarting exercising seemed to be more difficult after the 2^nd^ LD. Objective training data analysis showed a decrease of strength scores mostly during the 2^nd^ LD. Regarding the numerous benefits of an active lifestyle, supporting measures for maintaining PA, especially for strengthening of the muscles, should be more strongly promoted. In times or regions of limited access to sports facilities home-based training options should be promoted especially for winter months when outdoor activities decrease.
